# The influence of rare variants in circulating metabolic biomarkers

**DOI:** 10.1371/journal.pgen.1008605

**Published:** 2020-03-09

**Authors:** Fernando Riveros-Mckay, Clare Oliver-Williams, Savita Karthikeyan, Klaudia Walter, Kousik Kundu, Willem H. Ouwehand, David Roberts, Emanuele Di Angelantonio, Nicole Soranzo, John Danesh, INTERVAL Study, Eleanor Wheeler, Eleftheria Zeggini, Adam S. Butterworth, Inês Barroso

**Affiliations:** 1 Wellcome Sanger Institute, Cambridge, United Kingdom; 2 MRC/BHF Cardiovascular Epidemiology Unit, Department of Public Health and Primary Care, University of Cambridge, Cambridge, United Kingdom; 3 Homerton College, Cambridge, United Kingdom; 4 Department of Haematology, University of Cambridge, Cambridge Biomedical Campus, Cambridge, United Kingdom; 5 NHS Blood and Transplant, Cambridge Biomedical Campus, Cambridge, United Kingdom; 6 The National Institute for Health Research Blood and Transplant Research Unit (NIHR BTRU) in Donor Health and Genomics, Department of Public Health and Primary Care, University of Cambridge, Cambridge, United Kingdom; 7 NHS Blood and Transplant—Oxford Centre, Level 2, John Radcliffe Hospital, Oxford, United Kingdom; 8 Radcliffe Department of Medicine, University of Oxford, John Radcliffe Hospital, Oxford, United Kingdom; 9 British Heart Foundation Centre of Research Excellence, University of Cambridge, Cambridge, United Kingdom; 10 National Institute for Health Research Cambridge Biomedical Research Centre, University of Cambridge and Cambridge University Hospitals, Cambridge, United Kingdom; 11 Health Data Research UK Cambridge, Wellcome Genome Campus and University of Cambridge, Cambridge, United Kingdom; 12 MRC Epidemiology Unit, Wellcome Trust-MRC Institute of Metabolic Science, Addenbrooke's Hospital, Cambridge, United Kingdom; 13 Institute of Translational Genomics, Helmholtz Zentrum München—German Research Center for Environmental Health, Neuherberg, Germany; University of California San Francisco, UNITED STATES

## Abstract

Circulating metabolite levels are biomarkers for cardiovascular disease (CVD). Here we studied, association of rare variants and 226 serum lipoproteins, lipids and amino acids in 7,142 (discovery plus follow-up) healthy participants. We leveraged the information from multiple metabolite measurements on the same participants to improve discovery in rare variant association analyses for gene-based and gene-set tests by incorporating correlated metabolites as covariates in the validation stage. Gene-based analysis corrected for the effective number of tests performed, confirmed established associations at *APOB*, *APOC3*, *PAH*, *HAL* and *PCSK (p*<1.32x10^-7^) and identified novel gene-trait associations at a lower stringency threshold with *ACSL1*, *MYCN*, *FBXO36* and *B4GALNT3* (*p*<2.5x10^-6^). Regulation of the pyruvate dehydrogenase (PDH) complex was associated for the first time, in gene-set analyses also corrected for effective number of tests, with IDL and LDL parameters, as well as circulating cholesterol (*p*_*METASKAT*_<2.41x10^-6^). In conclusion, using an approach that leverages metabolite measurements obtained in the same participants, we identified novel loci and pathways involved in the regulation of these important metabolic biomarkers. As large-scale biobanks continue to amass sequencing and phenotypic information, analytical approaches such as ours will be useful to fully exploit the copious amounts of biological data generated in these efforts.

## Author summary

Cardiovascular diseases (CVD) are the number one cause of death globally. Various metabolic biomarkers including lipid and lipoprotein particles have been implicated as risk factors for the development of CVD. Understanding how these biomarkers are regulated can lead to increased understanding of CVD aetiology and the potential identification of drug targets. Here we take advantage of high resolution measurements on a large cohort of 7,142 healthy blood donors with whole-exome and whole-genome sequencing data to explore the influence of rare variation on circulating metabolic biomarker levels. Using a novel approach leveraging the information gained from various measurements on the same participants we are able to identify a novel biological pathway involved in the regulation of intermediate-density and low-density lipoproteins as well as circulating cholesterol, confirm various established gene associations and identify potential novel gene associations that merit further replication. This work highlights the advantages that can be gained by combining high resolution genotypic and phenotypic measurements in one large cohort.

## Introduction

Metabolic measurements reflect an individual’s endogenous biochemical processes and environmental exposures [1,2]. Many circulating lipids, lipoproteins and metabolites have been previously implicated in the development of cardiovascular disease (CVD) [3–6] or used as biomarkers for disease diagnosis or prognosis [7,8]. Understanding the genetic influence on circulating levels of these metabolic biomarkers can help us gain insight into the biological processes regulating these traits, lead to improved aetiological understanding of CVD and identify novel potential therapeutic drug targets. Notable examples of candidate drug targets with support from human genetics are *LDLR* [9,10], *APOB* [11,12] and *PCSK9* [13,14].

Genome-wide association studies (GWAS) focusing on traditionally measured lipid traits have greatly expanded our knowledge into lipid biology and to date more than 250 loci have been robustly associated with total cholesterol (TC), high-density lipoprotein cholesterol (HDL-C), low-density lipoprotein cholesterol (LDL-C), and/or triglycerides (TG) [15–23]. In addition to this, more detailed metabolic profiling using high resolution nuclear magnetic resonance (NMR) measurements has proven helpful to find additional lipid and small molecule metabolism-associated loci with smaller sample sizes, and to assess pleiotropic effects of previously established loci [24–26]. An example of this, is a novel link between the *LPA* locus and very-low-density lipoprotein (VLDL) metabolism (measured by high resolution NMR), with effect sizes twice as large as those found for traditionally measured lipid traits like LDL-C and TC, suggesting these measurements are better at capturing underlying biological processes in lipid metabolism than traditionally measured lipid traits [25]. In this same study, by constructing a genetic risk score using variants associated with lipoprotein(a) levels and using a Mendelian randomisation approach the authors were able to determine a causal link between increased lipoprotein(a) levels on overall lipoprotein metabolism [25].

Despite the at scale usage of exome arrays to capture low-frequency and rare coding variation contributing to lipid and amino acid metabolism [19–22,26], large-scale sequencing studies have the added value of assessing rare variation at single nucleotide resolution across the whole genome, or exome, including the detection of private variants which could have large effects on protein function. These approaches enabled, for example, the discovery of inactivating variants in key proteins which are models for drug target antagonism [27,28].

In this study, we examined the contribution of rare variation (MAF <1%) to 226 serum metabolic measurements in 3,741 participants with whole-exome sequence (WES) data and 3,401 participants with whole-genome sequence (WGS) data from the INTERVAL cohort, which consists of generally healthy blood donors residing in the UK.

## Results

### Gene-based analyses

The sample size of this study gave us limited power to detect novel single variant associations at rare variants (power 9.7% to find an association at *p*<5x10^-8^ with MAF 0.1% and beta = 1.1), but was well-powered for common variant analysis (power 86.41% to find an association at *p*<5x10^-8^ with MAF 1% and beta = 0.55). Therefore, single variant analyses only confirmed established associations at 34 unique loci after meta-analysis (N_discovery_ = 3,741, N_validation_ = 3,401, **S1 Table, S1 Fig**). Median correlation of betas for genome-wide significant hits between WES and WGS was 0.97.

We then sought to discover new gene-trait associations for 226 NMR metabolic biomarkers using rare-variant (MAF <1%) aggregate tests. For this analysis we used WES data from 3,741 healthy blood donors from the INTERVAL cohort as a discovery dataset (**Methods**). We performed two nested approaches to group rare variants; first just loss-of-function (LoF) variants and secondly, LoF variants plus variants predicted to be likely deleterious by their Mendelian Clinically Applicable Pathogenicity (M-CAP) score (M-CAP score >0.025) [29] (MCAP+LoF) (**Methods**). To try to minimise the inclusion of predicted deleterious missense variants with no phenotypic consequences, we restricted these variants to those with MAF<1%. As we expect the majority of LoF variants to have an effect on protein function, we did not filter by MAF (however ~97% of LoF variants tested had a MAF<1%). Genes were taken forward for validation if they reached an arbitrary threshold of *p*<5x10^-3^ in the discovery dataset (**S2 and S3 Tables**). Validation was performed using whole-genome sequence (WGS) data from 3,401 independent participants from the same cohort, and we present results from meta-analysis of discovery plus validation datasets (N = 7,142) that meet Bonferroni correction for the number of genes in the genome (0.05/20,000 genes), i.e. gene-level significance [30–32] (*p<*2.5x10^-6^, **Table 1, Methods**), without further adjustment for multiple traits. After meta-analysis, five genes (*APOB*, *APOC3*, *PCSK9*, *PAH*, *HAL*) were associated with 92 different traits with *p*<1.32x10^-7^, which is the stringent significance threshold after additionally correcting for the effective number of tested phenotypes (**Table 1, Methods, S1 Fig**). All five have been previously associated with their respective traits [24,33,34]. As previously suggested, we used correlated metabolic biomarkers as covariates to boost power [35,36]. These correlated biomarkers were selected for each outcome based on their phenotypic correlation in our dataset, the genetic correlation in publicly available datasets and the metabolic biomarker supergroup (**Methods**). However, to minimise the possible collider bias this could incur, we only did this at the validation stage. This was to ensure there was at least suggestive evidence for association in the discovery stage without using any metabolite as a covariate (**Methods**). This resulted in 99 traits where using other metabolic biomarkers as covariates was possible (**S4 Table**). As expected, we found a significant increase in the strength of the association signal (p-value) for traits when we used other correlated traits as covariates compared to the unadjusted tests [35,36], with the most notable example being a >30 order of magnitude increase in association strength for *PAH* and phenylalanine (**S2 and S3 Tables and S5 Table, Table 1**). In total, 32 of the known gene-trait associations met our stringent significance threshold (*p*<1.32x10^-7^) only after adjusting for correlated traits (**S2 and S3 Tables**).

**Table 1:** Genes significantly associated (*p*<2.5x10^-6^) with at least one trait in gene-based analyses focusing on loss-of-function (LoF) or predicted deleterious missense by M-CAP plus loss-of-function (MCAP+LoF). Genes that meet gene-level significance after adjusting for multiple phenotypes (*p*<1.32x10^-7^) are highlighted in bold. Top trait: trait with the smallest p-value after meta-analysis adjusting for correlated metabolites. p-value (covs): p-value of meta-analysis (WES+WGS) after adjusting for correlated metabolites for top trait. If NA, this analysis was not performed for this trait due to no metabolic biomarkers meeting the criteria to be included as covariates in meta-analysis. p-value (raw): p-value of meta-analysis without adjusting for correlated metabolites for top trait. N WES: number of tested variants in WES. N WGS: number of tested variants in WGS. AC = Allele count. N overlap: number of variants present in both WES and WGS. N traits associated: number of traits that meet gene-wide significance after adjusting for multiple phenotypes (*p*<1.32x10^-7^), traits meeting standard gene-wide significance (2.5x10^-6^) in parenthesis. Driven by single variant?: Yes if after conditioning on top associated variant the meta-analysis association disappears (*p*>0.05). IDL-TG: Triglycerides in IDL. XS-VLDL-TG: Triglycerides in very small VLDL. Phe: Phenylalanine. His: Histidine. IDL-FC: Free cholesterol in IDL. IDL-P: Concentration of IDL particles. M-VLDL-L: Total lipids in medium VLDL. Gly:Glycine. XL-HDL-FC: Free cholesterol in very large HDL. IDL-CE %: Cholesterol esters to total lipids ratio in IDL. L-VLDL-FC %: Free cholesterol to total lipids ratio in large VLDL. XXL-VLDL-C %: Total cholesterol to total lipids ratio in extremely large VLDL.

**LoF**										
**Gene**	**Top trait**	**p-value (covs)**	**p-value (raw)**	**N WES (AC)**	**p-value (WES)**	**N WGS (AC)**	**p-value (WGS)**	**N overlap**	**N traits associated**	**Driven by single variant?**
***APOB***	**IDL-TG**	**3.20x10^-13^**	**1.72x10^-10^**	**6 (6)**	**2.11x10^-10^**	**5 (9)**	**1.97x10^-3^**	**0**	**45 (57)**	**No**
***APOC3***	**XS-VLDL-TG**	**6.10x10-^13^**	**3.58x10^-12^**	**3 (18)**	**7.83x10^-6^**	**2 (23)**	**1.00x10^-7^**	**2**	**46 (56)**	**No**
**MCAP+LoF**										
**Gene**	**Top trait**	**p-value (covs)**	**p-value (raw)**	**N WES (AC)**	**p-value (WES)**	**N WGS (AC)**	**p-value (WGS)**	**N overlap**	**N traits associated**	**Driven by single variant?**
***PAH***	**Phe**	**8.33x10^-63^**	**1.67x10^-28^**	**39 (81)**	**1.93x10^-14^**	**41(79)**	**1.68x10^-14^**	**18**	**1 (1)**	**No**
***HAL***	**His**	**NA**	**3.72x10^-42^**	**48 (177)**	**5.65x10^-23^**	**37 (159)**	**7.80x10^-20^**	**22**	**1 (1)**	**No**
***APOC3***	**XS-VLDL-TG**	**5.46x10^-11^**	**2.15x10^-10^**	**6 (23)**	**1.72x10^-5^**	**6 (30)**	**2.91x10^-7^**	**3**	**26 (40)**	**No**
***PCSK9***	**IDL-FC**	**2.39x10^-10^**	**1.11x10^-7^**	**15 (38)**	**1.70x10^-4^**	**17 (33)**	**3.21x10^-5^**	**3**	**29 (34)**	**No**
*ACSL1*	IDL-P	1.82x10^-7^	1.76x10^-4^	4 (5)	4.52x10^-3^	6 (6)	2.68x10^-3^	2	0 (1)	Yes
*MYCN*	M-VLDL-L	6.20x10^-7^	3.97x10^-6^	7 (8)	8.25x10^-4^	8 (14)	7.44x10^-4^	3	0 (5)	No
*ALDH1L1*	Gly	NA	4.56x10^-7^	33 (132)	4.34x10^-5^	38 (128)	2.89x10^-3^	19	0 (1)	No
*SCARB1*	XL-HDL-FC%	NA	4.30x10^-7^	24 (38)	2.90x10^-4^	18 (40)	7.56x10^-4^	10	0 (6)	No
*FBXO36*	IDL-CE %	NA	1.98x10^-6^	5 (62)	1.62x10^-5^	2 (43)	2.56x10^-2^	1	0 (1)	Yes
*B4GALNT3*	L-VLDL-FC %	NA	7.59x10^-7^	27 (721)	1.07x10^-4^	22 (697)	1.61x10^-3^	13	0 (1)	No
*LIPC*	XXL-VLDL-C %	NA	9.04x10^-7^	27 (46)	1.94x10^-4^	29	2.53x10^-3^	11	0 (2)	No

In addition, we found 15 gene-trait associations in seven genes meeting standard gene-level significance before adjusting for multiple traits (*p<*2.5x10^-6^) which also had nominal evidence of association in the validation cohort (*p*<0.05). These associations also have an FDR adjusted p-value <0.3% after removing well known genes from the results list. Nine of these were gene-trait associations in three established genes (*ALDH1L1*, *SCARB1*, *LIPC*, **Table 1**), suggesting that other results achieving this significance threshold may warrant being prioritised for additional follow-up to establish their validity (in these genes the results are not driven by LoF variants, **S6 Table**).

For the four potentially novel genes associated with lipid traits, gene-trait analysis for LDL-C and triglycerides in UK Biobank participants with WES data provided suggestive evidence of association between LDL-C and *B4GALNT3* (p = 0.03) (**S7 Table**).

### Gene-set analyses

**Fig 1:**
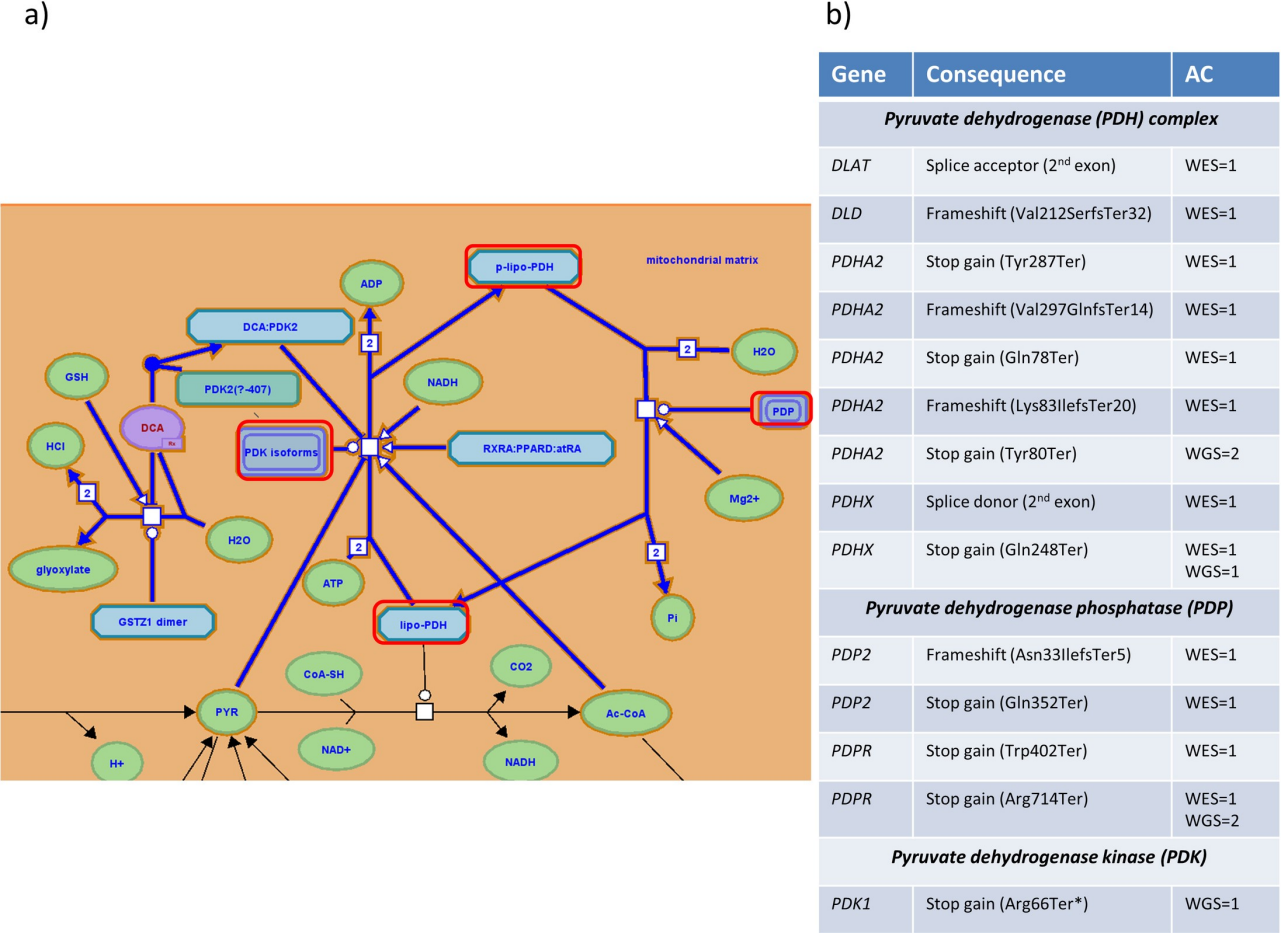
Loss-of-function (LoF) variants in regulation of pyruvate dehydrogenase (PDH) complex pathway. a) Figure adapted from REACTOME pathway browser [77]. Highlighted in red are protein complexes that carry LoF variants in INTERVAL WES or WGS. b) List of genes, consequences and allele count (AC) of LoF variants in the different protein complexes in the pathway.

To find links between predicted LoF variants and metabolic biomarker biology, we next explored associations of these variants in 7,150 gene-sets. To this end, we used two biological pathway databases (Reactome, KEGG) and one database that contains expert curated disease associated genes (DisGeNET) (**S8 Table, Methods**). Gene-set analysis yielded 163 gene-set-trait associations with 14 unique gene-sets meeting Bonferroni corrected gene-set-wide significance threshold (*p*_*meta*_<2.41x10^-6^, **Methods**, **S9 Table**). Given that 143 gene-set-trait associations were with 13 gene-sets that included two genes with well-established roles in lipid biology (*APOB* and *APOC3*), we repeated the test removing variants in these genes. After removal, there was residual evidence of association (*p*_*meta*_<0.05) in 102 of 143 gene-set-trait signals representing 12 of 13 gene-sets. Of the 163 gene-set-trait associations, the remaining 20 gene-set-trait associations (in gene-sets not containing either *APOB* or *APOC3*) represent associations of various lipoprotein-related metabolic biomarkers with the “regulation of pyruvate dehydrogenase (PDH) complex” pathway in REACTOME (R-HSA-204174, min *p* = 7.85x10^-7^, trait = phospholipids in intermediate density lipoproteins (IDL-PL), **S9 Table**). These associations encompassed 12 LoF (allele count [AC] = 12) variants in WES and four in WGS (AC = 6) (**Fig 1**). Upon further inspection, we found that the optimal rho(ρ) value in the SKAT-O test was one, in both the WES and the WGS analyses. This is equivalent to a burden test and suggests most variants tested in this pathway contribute to the association (i.e the signal was not driven by a single gene) [37] (**S10 Table**). Two variants were of particular interest as they were present in both WES and WGS datasets, rs113309941 in Pyruvate Dehydrogenase Complex Component X (*PDHX*) and rs201013643 in Pyruvate Dehydrogenase Phosphatase Regulatory Subunit (*PDPR*). In *PDHX*, rs113309941 leads to a premature stop mutation (Gln248Ter). It has an AC of one in both WES and WGS, and is very rare in the Genome Aggregation Database (gnomAD) (AC = 3, allele number (AN) = 246,116). rs201013643 in *PDRP* also leads to a premature stop (Arg714Ter) and is present in a single heterozygous participant in the WES dataset and two heterozygous participants in the WGS. This variant is also rare in gnomAD (AC = 141, AN = 275,988). The five participants carrying these two variants, who are all unrelated (PI_HAT<0.01) to carriers of the same variant, had higher than average values for biomarkers including cholesterol in intermediate-density lipoproteins (IDL-C) and LDL-C (lying in upper percentile range from 44.1% to 0.03% for both traits). Additionally, there are four unrelated heterozygous carriers of these variants in the European ancestry, UK Biobank participants with WES data (N = 36,769), of whom three had LDL-C measured at the baseline visit using a conventional enzymatic assay. Two of these individuals were in the upper quartile of LDL but the third one was in the bottom 1%” (**S11 Table**). These results suggest that the variants may have a deleterious but not fully penetrant impact on lipid metabolism, or that differences between the assay platforms across the studies may lead to heterogeneous associations. Future analyses of NMR assay measures in the UK Biobank may help to clarify this. None of the genes in this pathway has been previously associated with these traits and therefore this study links these genes collectively to IDL and LDL metabolism and circulating cholesterol for the first time.

### Enrichment of rare variant associations in genes near established GWAS signals in lipoprotein related metabolic biomarkers

Next, we conducted analyses to investigate whether genes near GWAS index variants associated with traditional lipid traits (HDL-C, LDL-C, TC and TG) were enriched for rare variant associations with high resolution lipoprotein measurements, which could suggest enrichment of effector transcripts in the gene-set. Given that this was a hypothesis-driven approach using established signals, to boost discovery power we pooled together both WES and WGS data into a single dataset of 7,142 participants. First, we extracted from the GWAS catalog (release 27-09-2017) the “reported genes” near signals that have been associated with HDL-C, LDL-C, TC or TG and created four gene-sets (**S12 Table**). We only focused on genes that were reported unambiguously (i.e. where only one gene is reported) since for associations where more than one gene is reported, it is possible that only one will be the effector gene and rare variants from the non-effector genes will only add noise to the analysis and therefore reduce power. We grouped rare variants in the gene-set using two nested approaches (LoF and MCAP+LoF) and ran SKAT-O on the gene-sets for 157 lipoprotein and lipid traits. Using this approach we found associations (*p*<0.005 correcting for effective number of tests, **Methods**) for genes near HDL-C GWAS signals with 18 HDL-related traits (**S13 Table**), the strongest association being with esterified cholesterol in extra-large HDL (XL-HDL-CE, *p* = 2.83x10^-5^, MCAP+LoF). Associations (*p*<0.005, **Methods**) in two XL-HDL-C related traits remained after removing variants in genes known to be involved in conditions leading to abnormal lipid levels or genes where functional work has shown an effect on HDL-C (**S14 Table, Methods**), and after conditioning on associated common variants near reported genes (XL-HDL-CE, *p* = 0.002 and XL-HDL-C, *p =* 0.004). These findings suggest that there is a contribution to the phenotypic variance of these traits by rare coding variants in genes near GWAS signals without a known role in HDL metabolism, which may represent novel effector transcripts.

### Enrichment of rare variation in tails of the phenotypic distribution of lipoprotein and glyceride related traits

Finally, we aimed to investigate whether participants at the extreme tails of the phenotype distribution for 106 lipoprotein and lipid traits harboured rare coding variants likely to be contributing to their phenotype. We used the WES dataset as a discovery dataset and the WGS dataset for validation. An arbitrary cut-off of 10 participants at each tail was used to define the tails for all of the 106 traits (**Methods**). After meta-analysis, we found an enrichment of deleterious rare variation (validation *p*_*permutation*_<0.05, meta-analysis *p*_*permutation*_<0.00037, **Methods, Table 2, S15 Table**) in hyperlipidaemia related genes in the lower tail of cholesterol in small VLDL (S-VLDL-C), esterified cholesterol in small VLDL (S-VLDL-CE) and concentration of extra small VLDL particles (XS-VLDL-P), and rare variation in HDL remodelling related genes in the lower tail of concentration of small HDL particles (S-HDL-P). We still observed nominal evidence of association in the WES and WGS datasets for the S-VLDL-C and XS-VLDL-P results using a 0.5% percentile cut-off for the tails but no evidence of association was found when using a 1% percentile cut-off (**S16 Table**). This is likely due to the fact that by increasing the number of participants taken from the tails, we are decreasing the average distance to the mean of the trait distribution and diluting signal coming from true extreme values.

**Table 2:** Gene-sets where there is a nominally significant enrichment of rare variation in the tails of a lipid or lipoprotein measurement (*p*>0.05) in both WES and WGS. **–** Highlighted in bold are gene-sets that are significant after meta-analysis using Stouffer’s method [38] and after adjusting for multiple traits (*p*< = 0.00037). WES P: permutation *p* in WES. WGS P: permutation *p* in WGS. Meta-P: *p* after meta-analysis using Stouffer’s method. S-VLDL-FC: Free cholesterol in small VLDL. XS-VLDL-C: Cholesterol in very small VLDL. S-VLDL-C: Cholesterol in small VLDL. XS-VLDL-P: Concentration of very small VLDL particles. S-VLDL-CE: Cholesterol esters in small VLDL. S-HDL-P: Concentration of small HDL particles.

**Upper tails**				
**Trait**	**WES P**	**WGS P**	**Meta-P**	**Gene-set**
S-VLDL-FC	3.3x10^-2^	2.37x10^-2^	3.45x10^-3^	Hypertriglyceridemia_HPO
XS-VLDL-C	3.3x10^-2^	2.37x10^-2^	3.45x10^-3^	Hypertriglyceridemia_HPO
**Lower tails**				
**Trait**	**WES P**	**WGS P**	**Meta-P**	**Gene-set**
**S-VLDL-C**	**5.8x10**^**-3**^	**2.31x10**^**-3**^	**7.61x10**^**-5**^	**Hyperlipidaemia**
**XS-VLDL-P**	**1.85x10**^**-2**^	**7x10**^**-4**^	**9.42x10**^**-5**^	**Hyperlipidaemia**
**S-VLDL-CE**	**5.8x10**^**-3**^	**6.75x10**^**-3**^	**2.07x10**^**-4**^	**Hyperlipidaemia**
**S-HDL-P**	**2.72x10**^**-3**^	**1.84x10**^**-2**^	**2.89x10**^**-4**^	**HDL_remodeling**
S-HDL-P	4.10x10^-2^	3.92x10^-2^	8.x24x10^-3^	Hypertriglyceridemia_CTD

## Discussion

Exploring rare coding variation provides an opportunity to gain insights into biological processes regulating the circulating levels of metabolic biomarkers. Here we take advantage of the combination of sequencing data and high-resolution NMR measurements to elucidate how this variation influences multiple metabolic measurements in a healthy cohort of UK blood donors.

To identify genes and gene-sets associated with metabolic biomarkers, we used a two-stage gene-based analysis using WES data for discovery (N_discovery_ = 3,741) and WGS data for validation (N_validation_ = 3,401). Rare-variant aggregation tests were used to identify genes harbouring multiple rare coding variants associated with metabolic biomarkers. To gain power at the validation stage we adjusted analyses for correlated traits, an approach previously described for single variant analysis [36]. This yielded significant power gains, notably for the known association of *PAH* with phenylalanine levels, where adjusting for 71 phenotypically correlated traits resulted in a greater than 30-fold magnitude change in the statistical evidence of association after meta-analysis. Overall, this approach yielded 4,114 gene-trait associations taken forward for validation (p_discovery_<5x10^-3^). After meta-analysis, besides recapitulating previous associations in eight known genes (*APOB*, *APOC3*, *PAH*, *HAL*, *PCSK9*, *ALDH1L1*, *SCARB1* and *LIPC*, **Table 1**), this method also identified four genes (*ACSL1*, *MYCN*, *B4GALNT3*, *FBXO36*) that met standard gene-level significance (*p*<2.5x10^-6^, **Table 1**) in at least one gene-trait association test. Of these, *ACSL1* and *MYCN* have been previously linked to lipid metabolism [39–41], and therefore will merit additional follow-up. Of these four genes only *B4GALNT3* had evidence of association with a traditional lipid trait (LDL-C, p<0.05) in UK Biobank although it is important to note that the lead associations in the INTERVAL study for these genes were with specific lipoprotein parameters and none of these genes except *ASCL1* (serum triglycerides, *p = 0*.*03*) showed evidence of association with a traditional lipid trait in the INTERVAL NMR data.

*ACSL1*, which codes for long-chain-fatty-acid—CoA ligase 1, is the predominant isoform of *ACSL* in the liver. The gene was associated with concentration of IDL particles in this study (*p* = 1.82x10^-7^), and its deficiency in the liver has been shown to reduce synthesis of triglycerides and beta oxidation, and alter the fatty acid composition of major phospholipids [42]. An intronic variant (rs60780116) in *ACSL1* has been associated with risk of Type 2 diabetes [43] and elevated expression of *ACSL1* has been shown to be an independent risk factor for acute myocardial infarction after taking into account conventional risk factors [44].

*MYCN* encodes N-myc proto-oncogene protein and its amplification can lead to tumorigenesis [45,46]. Previous animal studies have shown that inhibition of *MYCN* can lead to accumulation of intracellular lipid droplets in tumour cells [41]. Here we find association between *MYCN* and concentration of lipids, phospholipids and triglycerides in medium VLDL, total particle concentration of medium VLDL and triglycerides in small VLDL (min *p* = 6.20x10^-7,^ **Table 1, S3 Table**).

The other two genes do not have any obvious link to lipid metabolism. *B4GALNT3* encodes beta-1,4-N-acetyl-galactosaminyl transferase 3. This protein mediates the N,N'-diacetyllactosediamine formation on gastric mucosa [47]. Mouse knockouts have been associated with abnormal tail movements, abnormal retinal pigmentation and increased circulating alkaline phosphatase levels [48] and variants near the gene have been associated with height and hip circumference adjusted for BMI in human GWAS [49,50]. *FBXO36* is a member of the F-box protein family, a family known to be involved in protein ubiquitination [51]. Replication of these signals in additional studies would represent a novel link between these genes and lipid metabolism.

In gene-set analysis, the “regulation of pyruvate dehydrogenase (PDH) complex” pathway was newly associated with 20 traits, mostly related to IDL and LDL lipoproteins. None of the genes in this pathway have been previously linked to any of these phenotypes, and our data suggest the signal arises from a cumulative effect of LoF variants in different genes in the pathway (Fig 1), which represents a novel link between this pathway and lipoprotein metabolism. Notably, one of the carriers of the *PDHX* Gln248Ter variant was in the top 0.03% for LDL-C in the whole INTERVAL cohort (4.1 mmol/l, 158.0 mg/dl) and had no predicted deleterious missense mutations in known hypercholesterolemia genes (*PCSK9*, *APOB* or *LDLR*) suggesting this novel protein-truncating variant may be a genetic cause for their high LDL-C levels. The other carrier of this variant was in the top 19.3% percentile of the whole cohort (N = 46,083), but within the normal clinical range (1.8 mmol/l). Since we lack information on participants’ use of lipid-lowering medication, the degree to which this variant influences the observed LDL-C levels is difficult to assess. The PDH complex has been shown to be crucial for metabolic flexibility, i.e. the capacity to adjust fuel oxidation based on nutrient availability, which itself has been shown to play a role in cardiovascular disease [52].

In analyses aiming at identifying effector transcripts at established GWAS loci associated with traditional lipid measurements (HDL-C, LDL-C, TC and TG), we established that reported genes mapping near HDL-C associated loci were enriched for rare coding variants associated with multiple HDL-related measurements. The results remained significant (p<0.005) after removing genes known to be directly involved in HDL metabolism. This suggests that even though no single gene in the gene-set has sufficient statistical evidence of association, rare variants in this gene-set contribute to variation in these traits, and that this gene-set is enriched for additional effector transcripts.

Finally, we showed that one can detect enrichment of rare variation in genes involved in lipoprotein metabolism in phenotypic extremes of some of these NMR measurements. Specifically, we showed enrichment of rare variants in hyperlipidaemia related genes in participants with very low levels of cholesterol and esterified cholesterol in small VLDL, and very low levels of the total concentration of small VLDL particles. Enrichment of rare variants in HDL remodelling genes in participants with very low levels of small HDL particles was also observed. Given that high levels of small HDL particles have been previously associated with higher incidence of ischemic stroke [53] some of these variants could have protective effects. These results are in agreement with previous work on LDL-C [23] and HDL-C [54] that show that common polygenic signals seem to have a higher impact on the higher extremes of lipid traits whereas there is evidence for a higher prevalence of rare variation on the lower extremes [54]. This is also expected since the INTERVAL cohort consists of predominantly healthy blood donors and therefore might be depleted of individuals with rare “damaging” variants. Our study benefits from utilising a novel approach incorporating correlated metabolite information in rare variant association analyses to boost power under a discovery plus validation study design. This kind of approach will prove useful as we continue to amass deeply phenotyped and sequenced biobanks. Whereas other similar studies [20,26,55] have been more lenient and not accounted for the multiple phenotypes tested, here in gene-based analyses we attempted to overcome this limitation by accounting for the effective number of tests performed, acknowledging this burden of proof limited the power of our study.

One limitation of studies using sequencing data is that some of the singleton variants that contribute to the rare variant tests might be false positives as they have not been validated with a different molecular approach. We tried to overcome this limitation by using a two-stage design in our gene-based and gene-set analyses requiring evidence of association in the two independent datasets. This approach should decrease the possibility of false-positive findings as the likelihood that the same gene or gene-set would be a false-positive in both WES and WGS datasets is decreased. Additionally, for the novel association of the PDH pathway with multiple lipid traits, we manually inspected the depth of coverage for all variants tested and found that all of them were well supported with a min DP of 19 for the alternative allele in the WES dataset except for one participant with DP<9 for a doubleton in the WGS dataset (**S17 Table**).

Altogether, our results show that focusing on rare variation and deep metabolic phenotyping provides new insights into circulating metabolic biomarker biology. This argues for the expansion of deeper molecular phenotyping as part of large cohort sequencing efforts to gain further understanding of the role of rare coding variation on circulating metabolic biomarkers which may potentially lead to novel drug target discovery and/or provide additional genetic validation for specific targets.

## Methods

### Participants

The INTERVAL cohort consists of nearly 50,000 predominantly healthy blood donors in the UK [56]. All participants were genotyped using the Affymetrix UK Biobank Axiom Array and imputed using a combined UK10K-1000G Phase III imputation panel [57]. A subset of 4,502 participants was selected for whole-exome sequencing (WES) [58] and another subset of 3,762 was selected for whole-genome sequencing (WGS). There was an overlap of 54 participants in both datasets.

### Sequencing and genotype calling

WES and WGS were performed at the Wellcome Sanger Institute (WSI) sequencing facility. For WES, sheared DNA was prepared for Illumina paired-end sequencing and enriched for target regions using Agilent’s SureSelect Human All Exon V5 capture technology (Agilent Technologies; Santa Clara, California, USA). The exome capture library preparation was sequenced using the Illumina HiSeq 2000 platform as paired-end 75 bp reads. Reads were aligned to the GRCh37 human reference genome using BWA (v0.5.10) [59]. GATK HaplotypeCaller v3.4 [60] was used for variant calling and recalibration. For WGS, sheared DNA was prepared for Illumina paired-end sequencing. Sequencing was performed using the Illumina HiSeq X platform as paired-end 75 bp reads. Reads were aligned to the GRCh38 human reference genome using mostly BWA (v.0.7.12) although a subset of samples was aligned with v.0.7.13 or v.0.7.15. GATK HaplotypeCaller v3.5 was used for variant calling and recalibration. We extracted coordinates from the VCF files that mapped to regions targeted in the WES. We then used custom scripts to transform coordinates of variants to GRCh37 human reference.

### Sample QC

For WES data we filtered out samples based on the following criteria: i) withdrawn consent; ii) estimated contamination >3% according to the software VerifyBamID [61]; iii) sex inferred from genetic data different from sex supplied; iv) non-European samples after manual inspection of clustering in 1000G principal component analysis (PCA) and choosing cutoffs on the first 2 PCs; v) heterozygosity outliers (samples +/- 3 SD away from the mean number of heterozygous counts); vi) non-reference homozygosity outliers (samples +/- 3 SD away from the mean number of non-reference homozygous counts); vii) outlier Ti/TV rates (transition to transversion ratio +/- 3 SD away from the mean ratio); viii) excess singletons (number of singleton variants >3 SD from the cohort mean). After quality control 4,070 WES samples were kept. For WGS data we filtered out samples based on the following criteria: i) estimated contamination >2% according to software VerifyBamID; ii) non-reference discordance (NRD) with genotype data on the same samples >4%; iii) population outliers from PCA (PC1 >0 and minimum PC2); iv) heterozygosity outliers (samples +/- 3 SD away from the mean number of heterozygous counts); v) number of third-degree relatives (proportion IBD (PI_HAT) >0.125) >18, vi) overlap with WES. After quality control 3,670 WGS samples were kept. The QC in WGS was done to maximise sample retention while losing low quality ones building upon previous work with similar data [62].

In UK Biobank, we filtered out samples based on the following criteria: i) withdrawn consent; ii) sex-mismatch between the sex inferred from the genetic data and the reported sex; iii) non-European ancestry according to genetic principal components of ancestry; iv) relatedness (third degree relatives in the UK Biobank data set). After quality control 36,769 WES samples were kept.

### Variant QC

For variants with MAF>1% we used the following thresholds to exclude variants: i) VQSR: 99.90% tranche for WES and 99% tranche for WGS; ii) missingness >3%; iii) HWE p<1x10-5. For variants with MAF≤1% the following thresholds were used: i) VQSR: 99.90% tranche for WES, 99% tranche for WGS SNPs and 90% tranche for WGS indels; ii) GQ:<20 for SNPs and <60 for indels; iii) DP<2; iv) AB>15 & <80 for heterozygous variants. After genotype-level QC (GQ,DP,AB) only variants with <3% missingness were kept. 1,716,946 variants were kept in the final WES release and 1,724,250 in the final WGS release.

For the UK Biobank WES data, we used the FE (GATK) callset and checked the depth of coverage for the regions targeting the novel genes. We removed monomorphic variants, variants with call rate < 95% and those with Hardy-Weinberg Equilibrium p-value < 1x10^-15^.

### Phenotype QC

A total of 230 metabolic biomarkers were produced by the serum NMR metabolomics platform (Nightingale Health Ltd.) [63] on 46,097 samples in the INTERVAL cohort. Glucose, lactose, pyruvate and acetate were excluded initially due to unreliable measurements. Conjugated linoleic acid and conjugated linoleic acid to total fatty acid ratio were set to missing for 3585 samples showing signs of peroxidation. Creatinine levels were set to missing for 1993 samples with isopropyl alcohol signals. Glutamine levels were set to missing for 347 samples that showed signs of glutamine to glutamate degradation. Samples with more than 30% missingness or identified as EDTA plasma were removed. After this step, for each pair of related samples (PI_HAT>0.125) we kept only one, preferentially keeping samples with the lowest missingness in WES or lowest NRD in WGS. Phenotypes were rank-based inverse normalised for all participants. We then separately performed linear regression for WES and WGS adjusting for age, gender, centre, processing duration, month of donation and 10 PCs. Residuals from both linear regressions were used as the outcome variables in all subsequent analyses. After this final step we kept 3,741 samples in the WES dataset and 3,401 samples in the WGS dataset.

LDL-C, HDL-C, total cholesterol and triglycerides were measured using enzymatic assays on a Beckman Coulter AU5800. Phenotypes in UK Biobank were rank-based inverse normalised within aliquot and combined, removing participants on lipid-lowering therapy (UKBB data codes 6177_or_6153 coded as “Cholesterol lowering medication”).

### Gene-based analyses

Coding variant consequences were annotated with VEP [64] using Ensembl gene-set version 75 for the hg19/GRCh37 human genome assembly. Loss-of–function (LoF) variants were annotated with a VEP plugin: LOFTEE (https://github.com/konradjk/loftee). M-CAP scores were downloaded and we extracted all missense variants with AC> = 1 in the WES or WGS datasets [29]. Two different nested tests were used to group rare variants into testable gene units: predicted to be high confidence LoF by LOFTEE in any transcript of the gene, and the same LoF variants plus rare (MAF <1%) missense variants mapping to any transcript of the gene predicted to be likely deleterious by M-CAP (M-CAP score >0.025) (MCAP+LoF).

We performed rare-variant aggregation tests as implemented in the SKAT-O R package [37,65]. For the LoF tests, we performed a burden test (rho = 1) whereas for the MCAP+LoF tests we used the optimal unified approach (method =“optimal.adj”). Genes were taken forward for validation if *p*<5x10^-3^.

Corresponding MCAP+LoF rare-variant aggregation tests for the four novel genes (*ACSL1*, *MYCN*, *FBXO36* and *B4GALNT3*) were performed in the European ancestry, unrelated participants in UK Biobank, adjusting for age, sex, aliquot and 10 principal components. Missense variants in any transcript were annotated with M-CAP scores on build 38 using ANNOVAR[66].

Adjusting for correlated phenotypes can increase power in single point association analyses [36], therefore to increase power, we implemented a strategy to incorporate information from the multiple phenotypes measured in our dataset. To minimise chances of a false positive association we only adjusted for phenotypes as covariates at the validation stage ensuring evidence of association in discovery stage was present without adjustment for covariates. In order for a metabolic biomarker to be selected as a covariate in the validation stage, the following conditions had to be met: i) no evidence of genetic correlation (*p*>0.05) with outcome using publicly available summary statistics from Kettunen *et al* (2016) [25]; ii) phenotypic correlation in our dataset >10%; iii) not belonging to same metabolic biomarker supergroup as outcome (**S18 Table**). This approach resulted in 99 eligible NMR traits for which other traits could be used as covariates. METASKAT [67] was used to perform meta-analysis using the same parameters as in discovery. A signal was considered to replicate if: i) it met our Bonferroni corrected gene-level significance threshold (*p*<1.32x10^-7^); ii) >2 variants were tested; iii) it was nominally significant (*p*<0.05) in the unadjusted test for WGS (i.e. without adjusting for correlated traits). The Bonferroni corrected gene-level significance threshold was chosen after adjusting the standard gene-level significance threshold (2.5x10^-6^) for 19 PCs explaining >95% of the variance of 226 metabolic biomarkers, an approach previously used in similar studies using the same NMR platform [24,25].

To test if a single variant was driving an observed association, we performed leave-one-out analysis for all variants contributing to the test. An association was considered to be driven by a single variant if, after removing it the test resulted in a non-significant association (*p>0*.*05*).

### Gene-set analyses

To perform gene-set analysis we obtained a curated gene-disease list from DisGeNET [68,69] and gene lists of metabolic pathways from KEGG [70–72] and Reactome [73,74] **(S8 Table).** The gene-disease list obtained from DisGeNET, combines expert curated gene-disease associations from the following databases: a) CTD (Comparative Toxicogenomics Database); b) UNIPROT; c) ORPHANET (an online rare disease and orphan drug data base); d) PSYGENET (Psychiatric disorders Gene association NETwork); and e) HPO (Human Phenotype Ontology). We limited analysis to gene-sets with more than three genes. Finally we extracted loss-of-function variants from genes in the gene-sets and ran SKAT-O (method =“optimal.adj”) for each of the traits. Similarly to the gene-based analysis, we used WES data as discovery, and took signals forward for validation in WGS if *p*<0.01. Covariate selection for correlated traits was performed as described in the gene-based analysis. The Gene-set-wide significance threshold was calculated by first estimating the effective number of gene-sets tested given the high overlap amongst them. Using PCA we estimated that 1094 PCs explain >95% of the variance in gene-sets. The significance threshold was therefore calculated as: 0.05/(1094*19) = 2.41x10^-6^ where 19 corresponds to the effective number of phenotypes tested as described above. A signal was considered to replicate if after meta-analysis: i) it met Bonferroni corrected gene-set-wide significance threshold (*p*_*meta*_<2.41x10^-6^); ii) >2 variants were tested; iii) it was nominally significant (p_validation_<0.05) in the unadjusted test for WGS (i.e without adjusting for correlated traits).

### Genes near GWAS signals

GWAS catalog data files (release 27-09-2017) were downloaded from https://www.ebi.ac.uk/gwas/docs/file-downloads [75]. We focused on GWAS loci associated with HDL-C, LDL-C, TC and TG. We extracted all reported genes for GWAS loci that were associated at genome-wide significance (*p*<5x10^-8^) excluding cases where the “REPORTED GENE” value was: i) NR (not reported); ii) intergenic; iii) APO(APOE) cluster; iv) HLA-area (**S12 Table**). These variants were also further used as covariates in sensitivity analyses. For this analysis, we ran SKAT-O using the optimal unified approach (method =“optimal.adj”) on the four gene-sets (HDLC reported, LDLC reported, TC reported, TG reported, **S12 Table**). The list of genes known to be involved in conditions leading to abnormal lipid levels was created extracting relevant genes from the DisGeNET and Reactome gene lists. Afterwards, we conducted a manual review of the published literature to remove genes where functional work in mouse or human has revealed a direct role of the gene in HDL metabolism (**S12 Table**). The search terms used were “[gene name] loss of function HDL” and “[gene name] knockout HDL”. Significance threshold (*p*<0.005) was determined by correcting for 10 PCs explaining >95% of the variance of the traits used in this analysis.

### Tails analysis

For this analysis, we used all lipoprotein and lipid traits but excluded derived measures (lipid ratios) resulting in 106 traits **(S18 Table).** We focused on likely deleterious missense and loss-of-function variation in lipid metabolism and disease gene-sets (**S19 Table**) with an allele count <10 in each dataset. We chose an arbitrary cut-off of 10 participants with the highest and lowest values for the traits to define our tails for all 106 traits. Given the high phenotypic correlation of our traits, there was a high overlap of participants at the tails of the distributions (**S2 and S3 Figs**) so we removed traits that shared > = 8 participants with any other trait reducing the number of tested traits to 50. For each trait, total deleterious allele count from each gene-set for upper and lower tails was obtained and an empirical *p* was calculated by performing 10,000 permutations extracting 10 random participants from the phenotype distribution and counting the number of deleterious alleles from the gene-set. This was done separately for WES (discovery) and WGS (validation) and only those that were nominally significant (*p*_*permutation*_<0.05) in WES were meta-analysed. The significance threshold for the combined WES+WGS meta-analysis (*p*_*permutation*_ = 0.00037) was chosen by correcting for 9 PCs explaining >95% of the traits variance and 15 pathways. Enrichment was declared if the test reached this significance threshold after meta-analysis and if in addition. we observed *p*_*permutation*_<0.05 in the validation dataset. Meta-analysis was done using Stouffer’s method [38] as implemented in the metap package [76] in R.

### Ethics statement

After reading study leaflets and participating in a discussion with donor carer staff, eligible donors were asked to complete the trial consent form before giving a blood donation. The National Research Ethics Service approved (11/EE/0538) this study.

## Supporting information

S1 Fig P-value thresholds used in each analysis. Neff refers to effective N. Yellow boxes highlight analyses adjusted for correlated metabolites.
(TIF)

S2 Fig Overlap of top 10 participants in the tails of 106 lipid and lipoprotein traits. **Columns represent the top 10 participants of at least one trait.** Rows represent the 106 lipid and lipoprotein traits used in this analysis. A blue square represents presence of a participant in the top 10 participants for its respective trait.
(TIF)

S3 Fig Overlap of bottom 10 participants in the tails of 106 lipid and lipoprotein traits. **Columns represent the lower 10 participants of at least one trait.** Rows represent the 106 lipid and lipoprotein traits used in this analysis. A blue square represents presence of a participant in the lower 10 participants for its respective trait.
(TIF)

S1 Table Single point association analyses results. (XLSX)

S2 Table Gene-trait associations with p< 5x10-3 in discovery stage (LoF, N = 3,741), and their corresponding association results in validation (N = 3,401) and combined meta-analysis (N = 7,142). (XLSX)

S3 Table Gene-trait associations with p< 5x10-3 in discovery stage (MCAP+LoF, N = 3,741) and corresponding validation results (N = 3,401) and combined meta-analysis results (N = 7,142). (XLSX)

S4 Table Phenotypes where adjustment for correlated biomarkers as covariates was performed. (XLSX)

S5 Table Variants tested in significant gene-based results. (XLSX)

S6 Table Gene-trait associations for candidate novel loci using only LoF. (XLSX)

S7 Table LoF + MCAP SKAT-O gene-based results for LDL and triglycerides in the white, unrelated samples with whole-exome sequence data in UK Biobank. (XLSX)

S8 Table Gene sets used in gene set analyses. (XLSX)

S9 Table Gene set analyses results. (XLSX)

S10 Table Rho values in SKAT-O tests for "R-HSA-204174" associated traits. (XLSX)

S11 Table LDL measurements for white, unrelated participants in UK Biobank (N = 36,769) with whole-exome sequence data carring LoF variants rs113309941 or rs201013643. (XLSX)

S12 Table Gene sets used for enrichment of genes near GWAS signals analyses. (XLSX)

S13 Table Results for SKAT-O analysis on gene sets built from lists of genes near established GWAS loci using two nested approaches (LoF and MCAP+LoF). (XLSX)

S14 Table Sensitivity analysis for significant results (p<0.005) in SKAT-O analysis on gene sets built from lists of genes near established GWAS loci. (XLSX)

S15 Table Detailed results for gene sets with enriched rare variation in tails of lipoprotein traits. (XLSX)

S16 Table Sensitivity analyses for rare variant enrichment in tails analysis using different percentile cutoffs to define tails of the phenotypic distribution. (XLSX)

S17 Table Gene set variants DP. (XLSX)

S18 Table List of traits and analyses where they were used. (XLSX)

S19 Table List of gene sets used for tails analyses. (XLSX)
