# Supplementary figures and images for: The influence of rare variants in circulating metabolic biomarkers
Source: PLoS Genet. 2020 Mar 9;16(3):e1008605. doi: 10.1371/journal.pgen.1008605 (PMC7108731; doi:10.1371/journal.pgen.1008605)

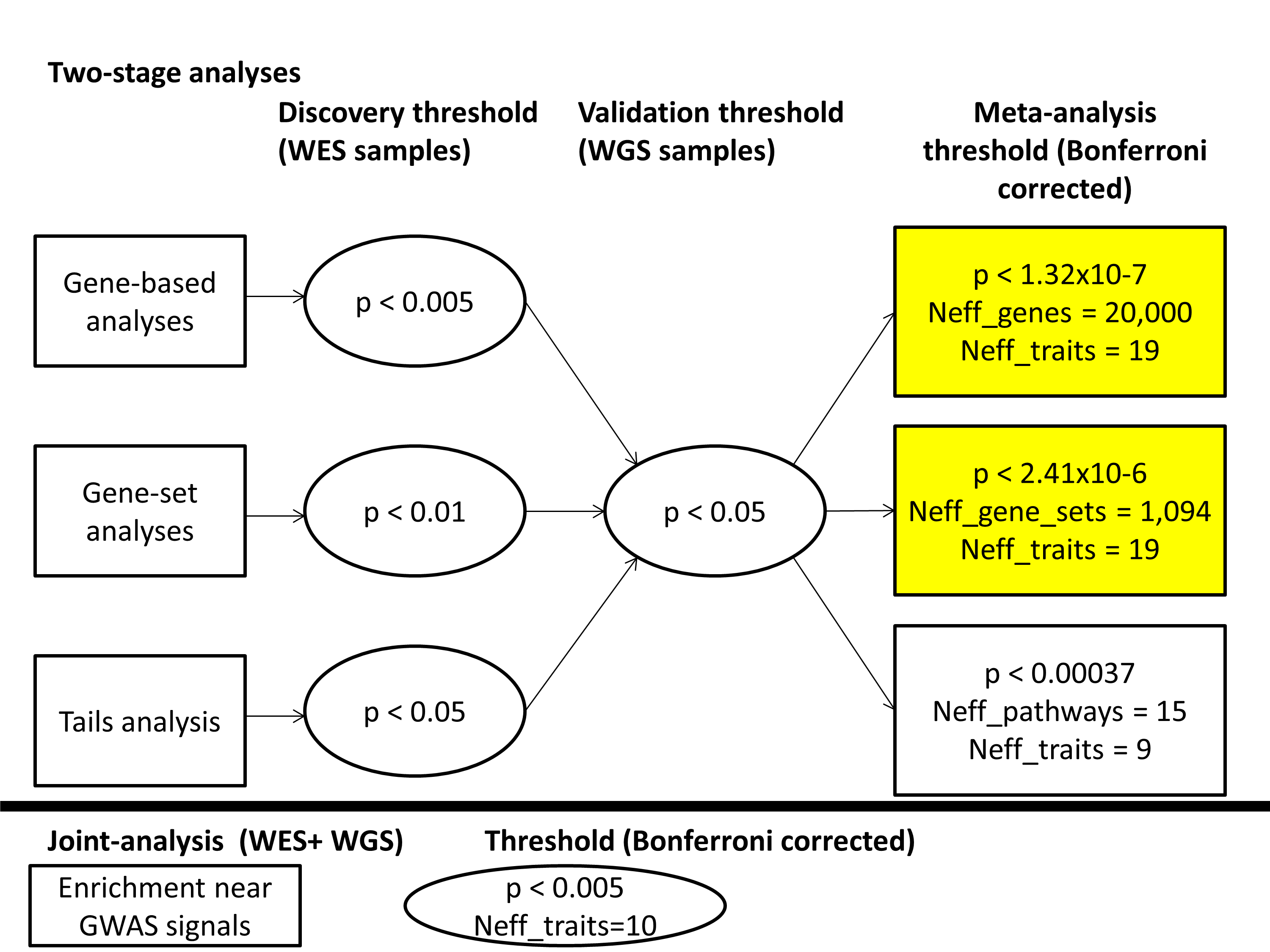

Supplement: S1 Fig — Neff refers to effective N. Yellow boxes highlight analyses adjusted for correlated metabolites. (TIF) [file pgen.1008605.s001.tif]

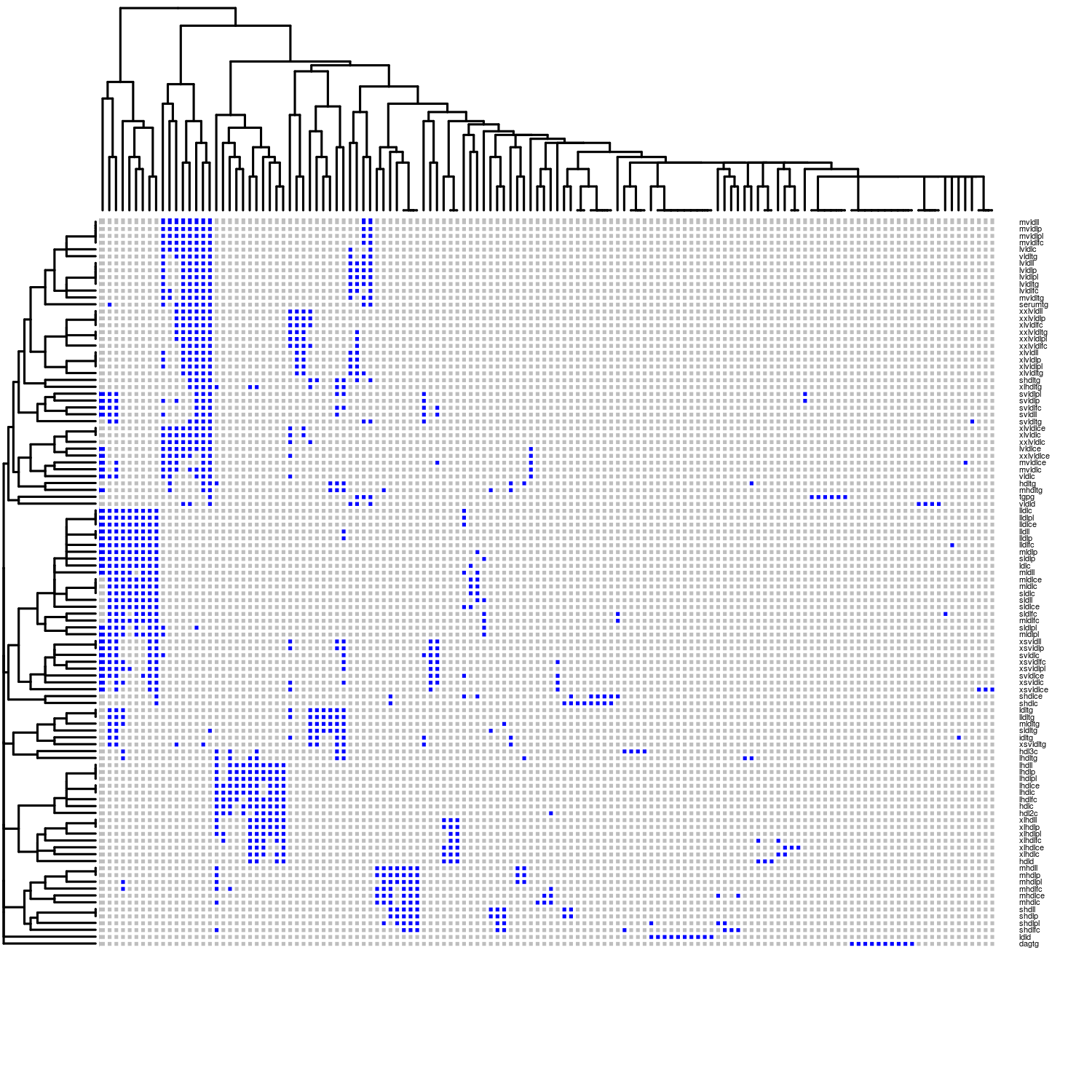

Supplement: S2 Fig — Columns represent the top 10 participants of at least one trait. Rows represent the 106 lipid and lipoprotein traits used in this analysis. A blue square represents presence of a participant in the top 10 participants for its respective trait. (TIF) [file pgen.1008605.s002.tif]

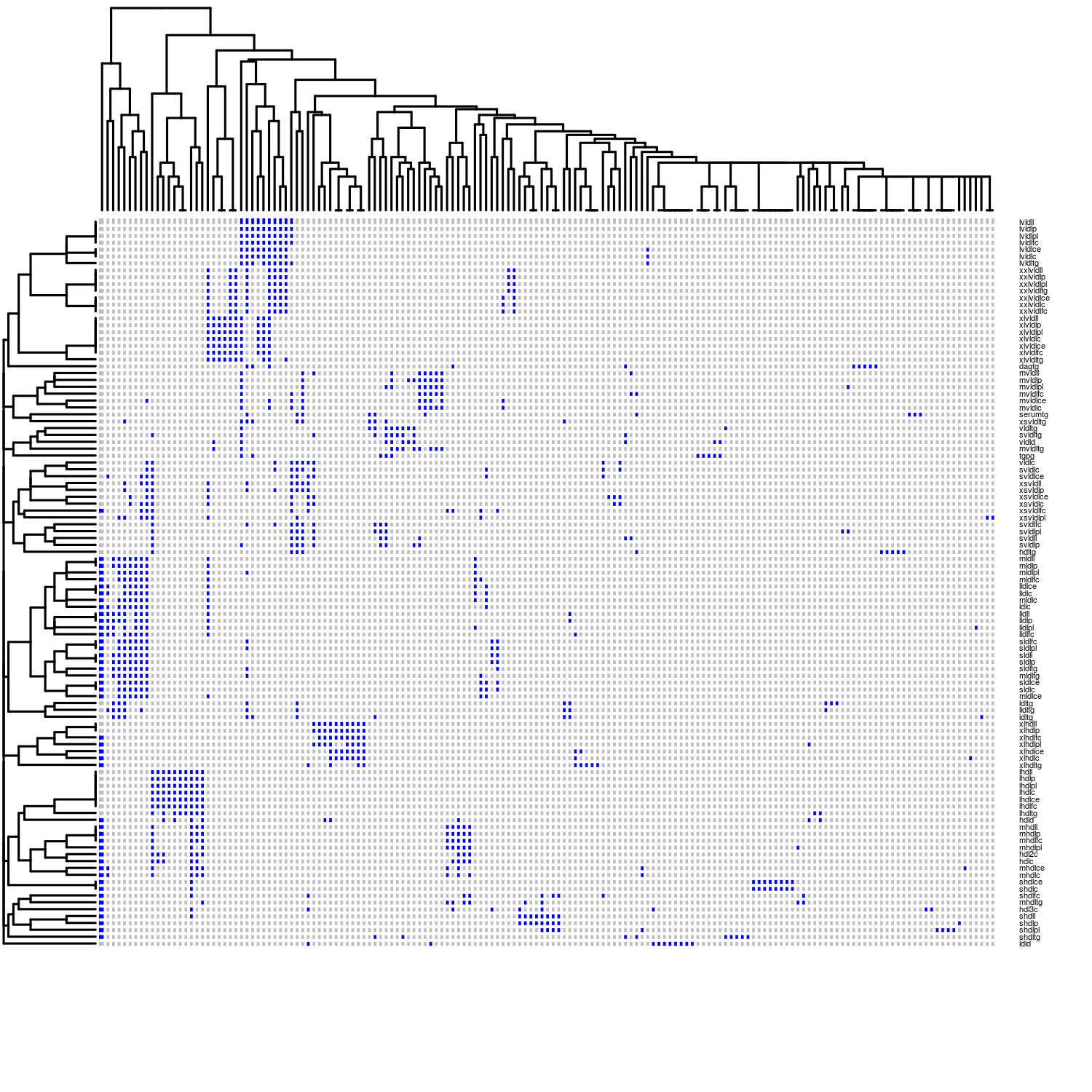

Supplement: S3 Fig — Columns represent the lower 10 participants of at least one trait. Rows represent the 106 lipid and lipoprotein traits used in this analysis. A blue square represents presence of a participant in the lower 10 participants for its respective trait. (TIF) [file pgen.1008605.s003.tif]
